# Involvement of RNA granule proteins in meiotic silencing by unpaired DNA

**DOI:** 10.1093/g3journal/jkab179

**Published:** 2021-05-13

**Authors:** Hua Xiao, Michael M Vierling, Rana F Kennedy, Erin C Boone, Logan M Decker, Victor T Sy, Jackson B Haynes, Michelle A Williams, Patrick K T Shiu

**Affiliations:** Division of Biological Sciences, University of Missouri, Columbia, MO 65211, USA; Division of Biological Sciences, University of Missouri, Columbia, MO 65211, USA; Division of Biological Sciences, University of Missouri, Columbia, MO 65211, USA; Division of Biological Sciences, University of Missouri, Columbia, MO 65211, USA; Division of Biological Sciences, University of Missouri, Columbia, MO 65211, USA; Division of Biological Sciences, University of Missouri, Columbia, MO 65211, USA; Division of Biological Sciences, University of Missouri, Columbia, MO 65211, USA; Present address: Department of Biology, McMaster University, Hamilton, ON L8S 4K1, Canada; Division of Biological Sciences, University of Missouri, Columbia, MO 65211, USA; Division of Biological Sciences, University of Missouri, Columbia, MO 65211, USA

**Keywords:** meiotic silencing by unpaired DNA (MSUD), *Neurospora crassa*, P-bodies, RNA granules, RNA interference (RNAi)

## Abstract

In *Neurospora crassa*, expression from an unpaired gene is suppressed by a mechanism known as meiotic silencing by unpaired DNA (MSUD). MSUD utilizes common RNA interference (RNAi) factors to silence target mRNAs. Here, we report that *Neurospora* CAR-1 and CGH-1, homologs of two *Caenorhabditis elegans* RNA granule components, are involved in MSUD. These fungal proteins are found in the perinuclear region and P-bodies, much like their worm counterparts. They interact with components of the meiotic silencing complex (MSC), including the SMS-2 Argonaute. This is the first time MSUD has been linked to RNA granule proteins.

## Introduction

*Neurospora crassa* grows as an interconnected network of tubular cells (hyphae). Because the cross walls between individual cells are usually incomplete, viruses and transposons can potentially infiltrate the entire fungal colony. To defend against these invasive elements, *Neurospora* maintains several genome surveillance systems (Aramayo and Selker 2013; Gladyshev 2017). One example is known as meiotic silencing by unpaired DNA (MSUD), an RNA interference (RNAi) mechanism that targets unpaired genes for silencing during sexual development (Shiu *et al.* 2001; Hammond 2017). This process begins at meiotic prophase I, when a gene without a pairing partner is detected (presumably with the help of the SAD-6 homology search protein; Samarajeewa *et al.* 2014). A single-stranded aberrant RNA (aRNA) is then produced from the unpaired region and exported out of the nucleus. In the perinuclear region, the aRNA is processed by the meiotic silencing complex (MSC), which contains several RNAi-related proteins (Decker *et al.* 2015). The SAD-1 RNA-directed RNA polymerase (RdRP), with the help of the SAD-3 helicase, converts the aRNA into a double-stranded RNA (dsRNA; Shiu and Metzenberg 2002; Hammond *et al.* 2011a). The DCL-1 Dicer cuts the dsRNA into small interfering RNAs (siRNAs), which are then made into single strands by the QIP exonuclease (Alexander *et al.* 2008; Xiao *et al.* 2010). The single-stranded siRNAs subsequently guide the SMS-2 Argonaute to target complementary mRNAs bound by nuclear cap-binding proteins NCBP1/2/3 (Lee *et al.* 2003; Decker *et al.* 2017; Boone *et al.* 2020). The SAD-2 scaffold protein functions to anchor many of the aforementioned factors to the perinuclear region (Shiu *et al.* 2006; Decker *et al.* 2015). SAD-4 and SAD-5 are required for siRNA production, although their precise roles in the pathway are currently unclear (Hammond *et al.* 2013a, 2013b). Finally, SAD-7 may coordinate nuclear and extranuclear silencing events (Samarajeewa *et al.* 2017).

Made up of RNA-protein aggregates, processing bodies (P-bodies) are cytoplasmic granules associated with the regulation of RNA translation, storage, and degradation (Jain and Parker 2013; Corbet and Parker 2019). Although P-bodies are enriched in proteins involved in translation repression and mRNA decay, the exact role of these structures remains to be determined (Luo *et al.* 2018; Tibble *et al.* 2021). In mammals, it has been suggested that mRNA decay may not take place inside P-bodies (Standart and Weil 2018), *i.e.*, P-bodies may function primarily to store mRNAs for later translation or decay (Riggs *et al.* 2020; Borbolis and Syntichaki 2021). In addition to the above, P-bodies have also been associated with RNAi (Jakymiw *et al.* 2007; Standart and Weil 2018). In this investigation, we explored the possibility that P-bodies are present during sexual development in *Neurospora* and asked whether some of their components are involved in MSUD.

## Materials and methods

### Fungal methods and genotypic information

Standard fungal protocols were followed throughout this study (http://www.fgsc.net/Neurospora/NeurosporaProtocolGuide.htm). Genotypes of *Neurospora* strains used are provided in Table 1. Genetic markers and knockout mutants are originally from the Fungal Genetics Stock Center (FGSC; McCluskey *et al.* 2010) and the Neurospora Functional Genomics Group (Colot *et al.* 2006). Culturing and crossing media were prepared according to Vogel (1956) and Westergaard and Mitchell (1947), respectively.

**Table 1:** *Neurospora* strains used in this study

Strain	Genotype
F2-01	*fl A* (FGSC 4317)
F2-29	*rid r* ^Δ^ *::hph; fl A*
F5-36	*fl; sad-5* ^Δ^ *::hph a*
F7-18	*fl; cgh-1* ^Δ^ *::hph A*
F8-01	*car-1* ^Δ^ *::hph fl A*
F8-10	*rid; fl; mCherry-dcap-2::hph; mus-51* ^Δ^ *::bar A*
F8-11	*rid; fl; mCherry-dcap-2::hph; mus-51* ^Δ^ *::bar; sad-5* ^Δ^ *::hph A*
F9-01	*fl; mCherry-dcap-2::hph; mus-51* ^Δ^ *::bar; cgh-1* ^Δ^ *::hph a*
P3-25	*mep sad-1* ^Δ^ *::hph a*
P9-42	Oak Ridge wild type (WT) *a* (FGSC 2490)
P11-67	*cgh-1* ^Δ^ *::hph a*
P17-70	*r* ^Δ^ *::hph; sad-5* ^Δ^ *::hph A*
P24-40	*rid; mCherry-dcap-2::hph; mus-51* ^Δ^ *::bar; gfp-cgh-1::hph A*
P24-41	*rid his-3; mCherry-dcap-2::hph; gfp-cgh-1::hph a*
P24-42	*rid; gfp-car-1::hph; mCherry-dcap-2::hph; mus-51* ^Δ^ *::bar A*
P24-43	*rid his-3; gfp-car-1::hph; mCherry-dcap-2::hph; mus-51* ^Δ^ *::bar a*
P24-64	*car-1* ^Δ^ *::hph a*
P25-19	*rid; gfp-car-1::hph; mCherry-cgh-1::nat A*
P25-20	*rid his-3; gfp-car-1::hph; mCherry-cgh-1::nat a*
P25-21	*rid; yfpc-car-1::hph; yfpn-cgh-1::nat A*
P25-22	*rid; yfpc-car-1::hph; yfpn-cgh-1::nat a*
P25-27	*rid; yfpc-car-1::hph; yfpn-sms-2::hph a*
P25-28	*rid; yfpc-car-1::hph A*
P25-29	*rid; yfpc-sms-2::hph yfpn-cgh-1::nat a*
P25-30	*rid; yfpc-sms-2::hph yfpn-cgh-1::nat A*
P25-31	*rid; yfpc-car-1::hph A*
P25-32	*rid; yfpc-car-1::hph; mus-51* ^Δ^ *::bar; yfpn-sad-2::hph a*
P26-18	*rid yfpc-sad-1::hph his-3; mus-51* ^Δ^ *::bar; yfpn-cgh-1::nat a*
P26-19	*rid yfpc-sad-1::hph; mus-51* ^Δ^ *::bar; yfpn-cgh-1::nat A*
P26-20	*rid his-3; mus-51* ^Δ^ *::bar yfpc-dcl-1::hph; yfpn-cgh-1::nat a*
P26-21	*rid; mus-51* ^Δ^ *::bar yfpc-dcl-1::hph; yfpn-cgh-1::nat A*
P26-22	*rid; yfpc-car-1::hph; mus-52* ^Δ^ *::bar; yfpn-dcl-1::nat a*
P26-23	*rid his-3; yfpc-car-1::hph; yfpn-dcl-1::nat A*
P26-24	*rid; yfpc-car-1::hph; yfpn-qip::nat mus-52* ^Δ^ *::bar a*
P26-25	*rid his-3; yfpc-car-1::hph; yfpn-qip::nat mus-52* ^Δ^ *::bar A*
P26-26	*rid yfpn-sad-1::nat; yfpc-car-1::hph a*
P26-27	*rid yfpn-sad-1::nat his-3; yfpc-car-1::hph A*
P26-28	*rid; mus-51* ^Δ^ *::bar; yfpc-sad-2::hph; yfpn-cgh-1::nat A*
P26-29	*rid; mus-51* ^Δ^ *::bar; yfpc-sad-2::hph; yfpn-cgh-1::nat a*
P26-30	*rid; yfpc-qip::hph; mus-51* ^Δ^ *::bar; yfpn-cgh-1::nat a*
P26-31	*rid; yfpc-qip::hph; mus-51* ^Δ^ *::bar; yfpn-cgh-1::nat A*
P26-32	*r* ^Δ^ *::hph; cgh-1* ^Δ^ *::hph a*
P26-34	*r* ^Δ^ *::hph; car-1* ^Δ^ *::hph a*
P26-36	*mCherry-dcap-2::hph a*
P26-37	*rid; mCherry-dcap-2::hph; mus-51* ^Δ^ *::bar; sad-5* ^Δ^ *::hph a*
P27-18	*car-1* ^Δ^ *::hph; mCherry-dcap-2::hph a*
P27-19	*rid; car-1* ^Δ^ *::hph; mCherry-dcap-2::hph; mus-51* ^Δ^ *::bar A*
P27-20	*rid; mCherry-dcap-2::hph; mus-51* ^Δ^ *::bar; cgh-1* ^Δ^ *::hph A*

Genetic loci are described in the *Neurospora crassa* e-Compendium (http://www.bioinformatics.leeds.ac.uk/∼gen6ar/newgenelist/genes/gene_list.htm).

### Transcript analysis

For comparison of gene expression, *Neurospora* vegetative (SRR080688, SRR081479, SRR081546, and SRR081586) and sexual (SRR957218) RNA-seq datasets were obtained from the European Bioinformatics Institute (EBI)’s European Nucleotide Archive (ENA) (Ellison *et al.* 2011; Samarajeewa *et al.* 2014). Computational analysis of these datasets was performed as described (Decker *et al.* 2017).

### Quantification of sexual spore production

*fluffy* (*fl*) strains were grown for six days in 24-well microplates (Corning #3524) at room temperature for use as designated females. Conidia (asexual spores) from each male strain were suspended in sterile water and adjusted to a concentration of 1000 counts/µl. For fertilization, 50 µl conidial suspension was inoculated on the female strain within a well. Ascospores (sexual spores) were collected from the lids 21 days post-fertilization and counted on a hemocytometer.

### MSUD assays

Most assessments of MSUD proficiency were performed according to the method of Xiao *et al.* (2019), with crosses conducted in 24-well microplates and analyses based on shot ascospores. For *cgh-1-*null crosses, sexual development is too impaired for proficient spore shooting. Accordingly, spores from these crosses were extracted out of the fruiting bodies for progeny phenotyping.

### Strain construction and confirmation

Green fluorescent protein (GFP) and mCherry tagging vectors were constructed using double-joint polymerase chain reaction (DJ-PCR; Hammond *et al.* 2011b; Samarajeewa *et al.* 2014). For strain confirmation, genomic DNA was isolated from conidia (Henderson *et al.* 2005) or vegetative hyphae (Qiagen DNeasy Plant Mini Kit). PCR-based validation of genotypes was conducted using the Promega GoTaq Green Master Mix or the Roche Expand Long Range dNTPack. When necessary, DNA sequencing was conducted by the University of Missouri (MU) DNA Core. Primers for strain construction and confirmation are listed in Supplementary Table S1.

### Bimolecular fluorescence complementation

Bimolecular fluorescence complementation (BiFC) is an *in vivo* assay to detect protein–protein interaction. In BiFC, a functional fluorophore is restored when two halves of the yellow fluorescent protein (YFP) are brought together by the association of two interacting proteins (Hu *et al.* 2002; Bardiya *et al.* 2008). Tagging of YFP halves was performed as previously reported (Hammond *et al.* 2011b).

### Photography and microscopy methods

Light microscopy images of protoperithecia (female structures), perithecia (fruiting bodies), and asci (spore sacs) were captured according to the methods of Decker *et al.* (2017). For fluorescent microscopy, a Leica TCS SP8 system was used. Preparation and visualization of asci were essentially as described (Alexander *et al.* 2008; Xiao *et al.* 2010).

### Image analysis of P-bodies

Images of asci in the single-nucleus (diploid) stage were analyzed with Fiji v2.0.0-rc-69-1.52p (Schindelin *et al.* 2012). The mCherry-DCAP-2 signal was used as a marker for P-bodies. After using the “Set Scale” function to input the pixel/μm ratio for an image, the number and average size (area) of P-bodies were obtained with the “Analyze Particles” function. For each cross, 8–24 asci were analyzed. The *P*-values were calculated using the two-tailed Student’s *t*-test.

## Results

### Presence of P-bodies in *Neurospora* meiotic cells

**Figure 1:**
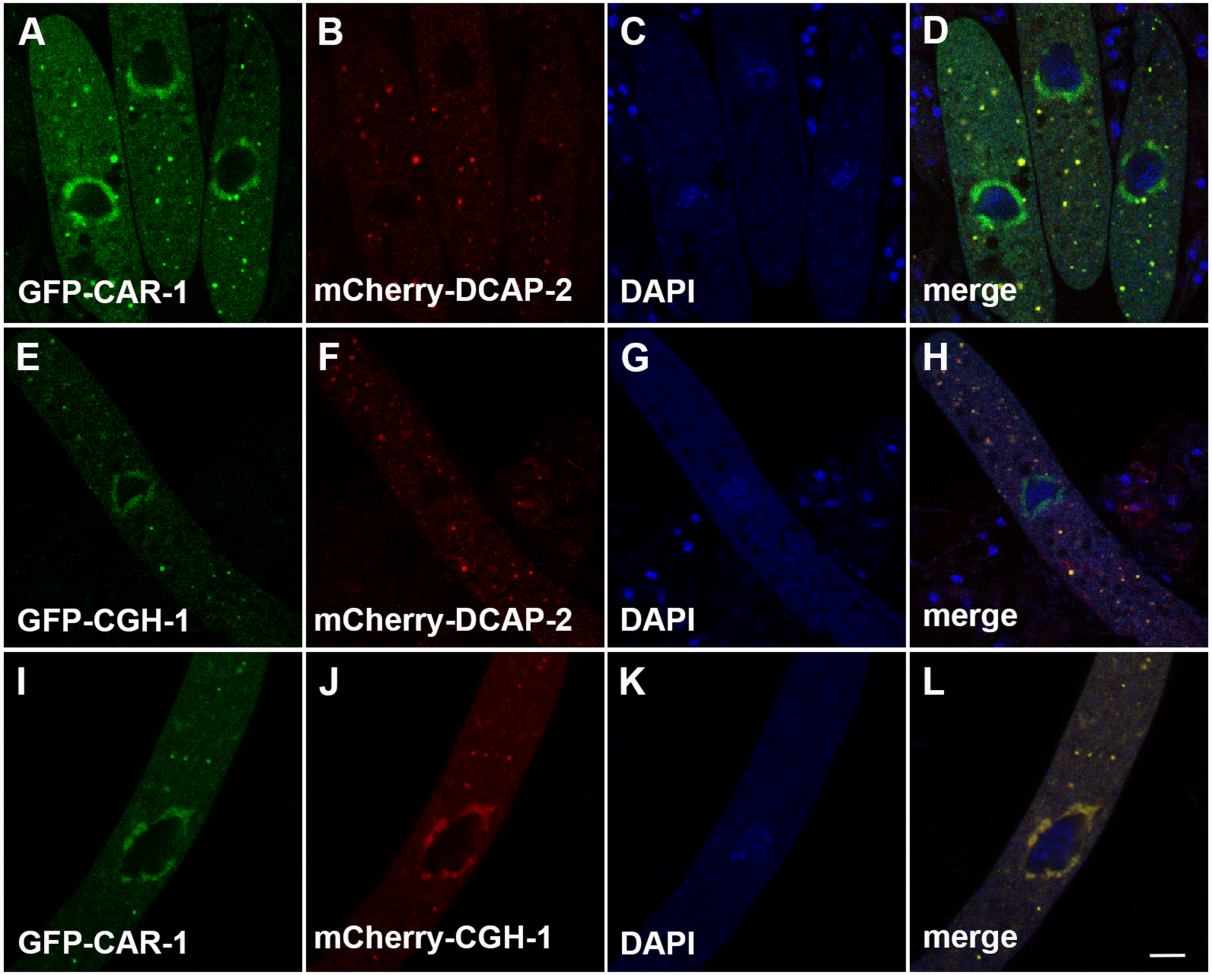
*Neurospora* CAR-1 and CGH-1 colocalize in the perinuclear region and P-bodies. DCAP-2 (decapping enzyme) is a P-body marker, and it colocalizes with cytoplasmic foci of CAR-1 and CGH-1 (D and H). Micrographs illustrate prophase asci expressing (A–D) *gfp-car-1* and *mCherry-dcap-2* (P24-42 × P24-43), (E–H) *gfp-cgh-1* and *mCherry-dcap-2* (P24-40 × P24-41), and (I–L) *gfp-car-1* and *mCherry-cgh-1* (P25-19 × P25-20). The chromatin was stained with DAPI. Bar, 5 μm.

We asked if P-bodies are present during the sexual phase of *Neurospora*. The DCAP-2 decapping enzyme is often used as a cellular marker for P-bodies (Sheth and Parker 2003; Gallo *et al.* 2008). Using mCherry-DCAP-2 (NCU07889), we have demonstrated that P-bodies can be seen in the asci (Figure 1B). To our knowledge, this is the first published P-body marker for *Neurospora*.

### CAR-1 and CGH-1 localize in the perinuclear region and P-bodies

CAR-1 (Sm-domain protein) and CGH-1 (RNA helicase) family proteins are conserved P-body components (Jain and Parker, 2013). In *Caenorhabditis elegans*, they are also parts of germline P granules (Updike and Strome 2010; Sundby *et al.* 2021). We have identified the corresponding homologs in *Neurospora* (NCU03366 and NCU06149) and tagged them with GFP. As seen in Figure 1, A–H, CAR-1 and CGH-1 localize in both the perinuclear region and P-bodies. This is reminiscent of the observations in worms, in which CAR-1 and CGH-1 are found in both perinuclear P granules and cytoplasmic P-bodies (Updike and Strome 2010; Jain and Parker 2013; Ko *et al.* 2013).

### CAR-1 and CGH-1 interact in P-bodies

**Figure 2:**
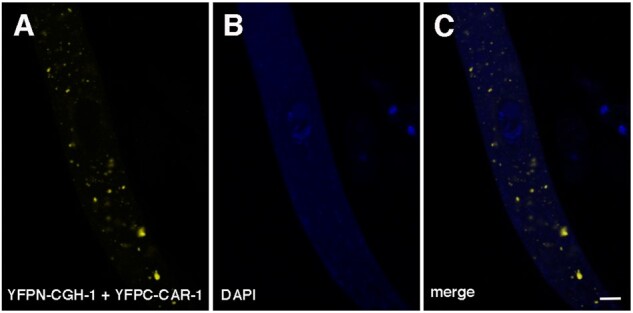
CAR-1 and CGH-1 interact in P-bodies. Interaction between tagged CAR-1 and CGH-1 allows the formation of a functional YFP (yellow) fluorophore. Micrographs illustrate prophase asci expressing *yfpn-cgh-1* and *yfpc-car-1* (P25-21 × P25-22). Bar, 5 μm.

CAR-1 and CGH-1 family proteins are known to have direct or indirect interaction with each other (Decker and Parker 2006; Roy and Rajyaguru 2018). Because *Neurospora* CAR-1 and CGH-1 colocalize (Figure 1L), we tested whether they actually have physical association. Using BiFC, we have detected interaction between CAR-1 and CGH-1 in cytoplasmic P-bodies and not in the perinuclear region (Figure 2). It is possible that they do not interact in the perinuclear region at all or that their interaction there is indirect (*e.g.*, through another protein).

### Expression of *car-1* and *cgh-1* during both vegetative and sexual stages

To determine the expression profiles of *car-1* and *cgh-1*, we examined their transcript levels through RNA-seq datasets. While presumed MSUD-exclusive genes have relatively low vegetative expression (as compared to their sexual expression), *car-1* and *cgh-1* are abundantly expressed in both developmental stages (Table 2). These results hint that the two RNA granule proteins are probably active in both sexual and asexual cycles.

**Table 2:** Expression of RNA silencing genes

Gene name	Gene no.	Vegetative expression (FPKM)	Sexual expression (FPKM)
RNA granules			
*car-1*	*ncu03366*	98.7509	65.0277
*cgh-1*	*ncu06149*	34.2955	72.2677
Housekeeping			
*actin*	*ncu04173*	2638.3425	905.4051
MSUD			
*sad-1*	*ncu02178*	0.3684	14.4495
*sad-2*	*ncu04294*	0.0000	38.5137
*sad-5*	*ncu06147*	0.0000	13.2559
*sms-2*	*ncu09434*	0.0496	673.0190
MSUD/Quelling			
*dcl-1*	*ncu08270*	4.4300	31.0978
*qip*	*ncu00076*	18.6841	107.2514

Quelling and MSUD refer to the vegetative and sexual silencing systems in *Neurospora*, respectively (Gladyshev 2017). FPKM, fragments per kilobase of exon per million mapped reads.

### *car-1* ^Δ^ and *cgh-1*^Δ^ mutants are slow growers

**Figure 3:**
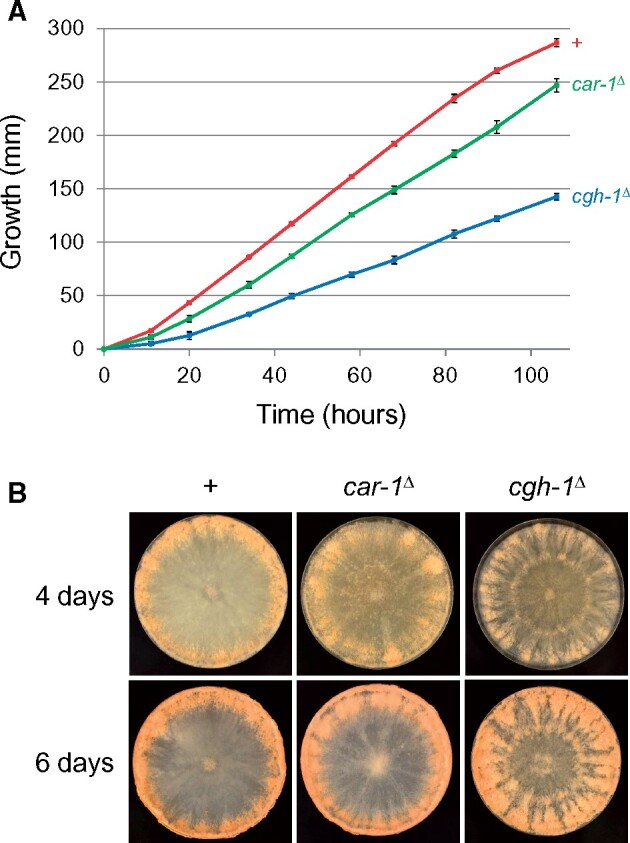
Vegetative phenotypes of *car-1* and *cgh-1* mutants. (A) Mutation in *car-1* (P24-64) or *cgh-1* (P11-67) leads to slower linear growth of the fungus, as compared to that of a + strain (P9-42). (B) For a *cgh-1*^Δ^ strain, dense conidiation is not limited to the perimeter of an agar plate. +, wild type (WT) at pertinent loci.

Deletion of the *car-1* or *cgh-1* homolog in yeast (*SCD6*/*DHH1*) is not lethal to the fungus (Hata *et al.* 1998; Kolesnikova *et al.* 2013). While *Neurospora car-1* and *cgh-1* are also nonessential, their losses are associated with slower linear growth (Figure 3A). Interestingly, deletion of *cgh-1* leads to an abnormal conidiation pattern (Figure 3B), suggesting its possible involvement in asexual development.

### Loss of CAR-1 or CGH-1 impairs sexual development

**Figure 4:**
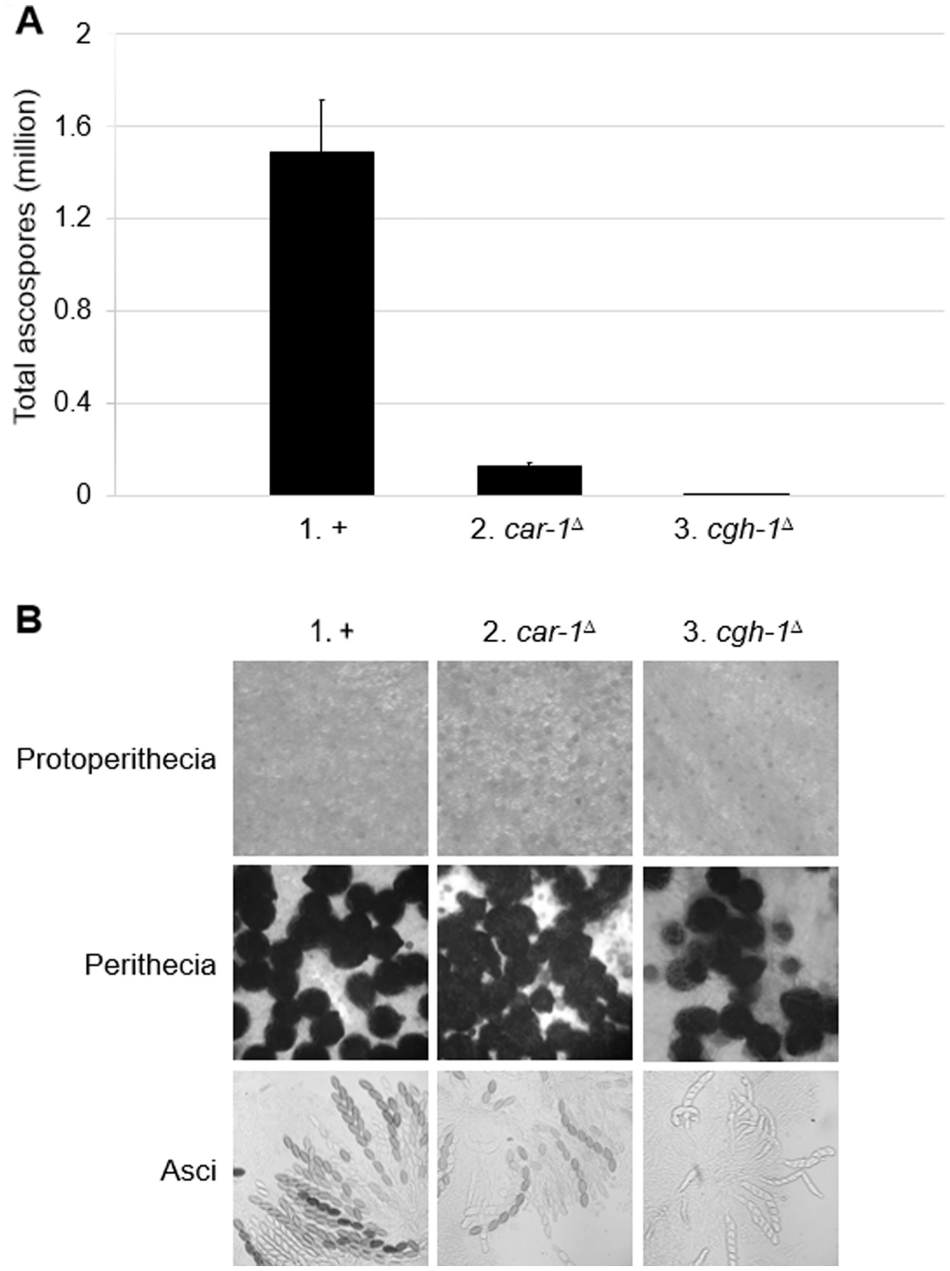
Crosses homozygous for *car-1*^Δ^ or *cgh-1*^Δ^ have defective sexual development. (A) Crosses missing *car-1* or *cgh-1* produce only a fraction of the spores seen in a normal cross (9 and 0.01%, respectively). (B) Although protoperithecial development appears proficient in *car-1*^Δ^ and *cgh-1*^Δ^ crosses, they only produce three-quarters and two-fifths of the normal number of mature perithecia, respectively. Dissected perithecia from these mutant crosses exhibit frequent and rampant ascus abortions, respectively. (1) F2-01 × P9-42. (2) F8-01 × P24-64. (3) F7-18 × P11-67.

Because *car-1* and *cgh-1* are well expressed during sexual development, we asked if they are required for ascospore formation. Although crosses homozygous for *car-1*^**Δ**^ or *cgh-1*^**Δ**^ are not completely barren, they typically produce only 9 and 0.01% of the normal number of spores, respectively (Figure 4A). Examination of the mutant crosses showed that they are deficient in perithecial and ascus development (Figure 4B). These results demonstrate that normal sexual reproduction in *Neurospora* requires CAR-1 and CGH-1, similar to the case in worms (Audhya *et al.* 2005). In yeast, deletion of the *car-1* homolog (*DHH1*) also leads to severe mating defects (Ka *et al.* 2008).

### CAR-1 and CGH-1 are important for MSUD

**Figure 5:**
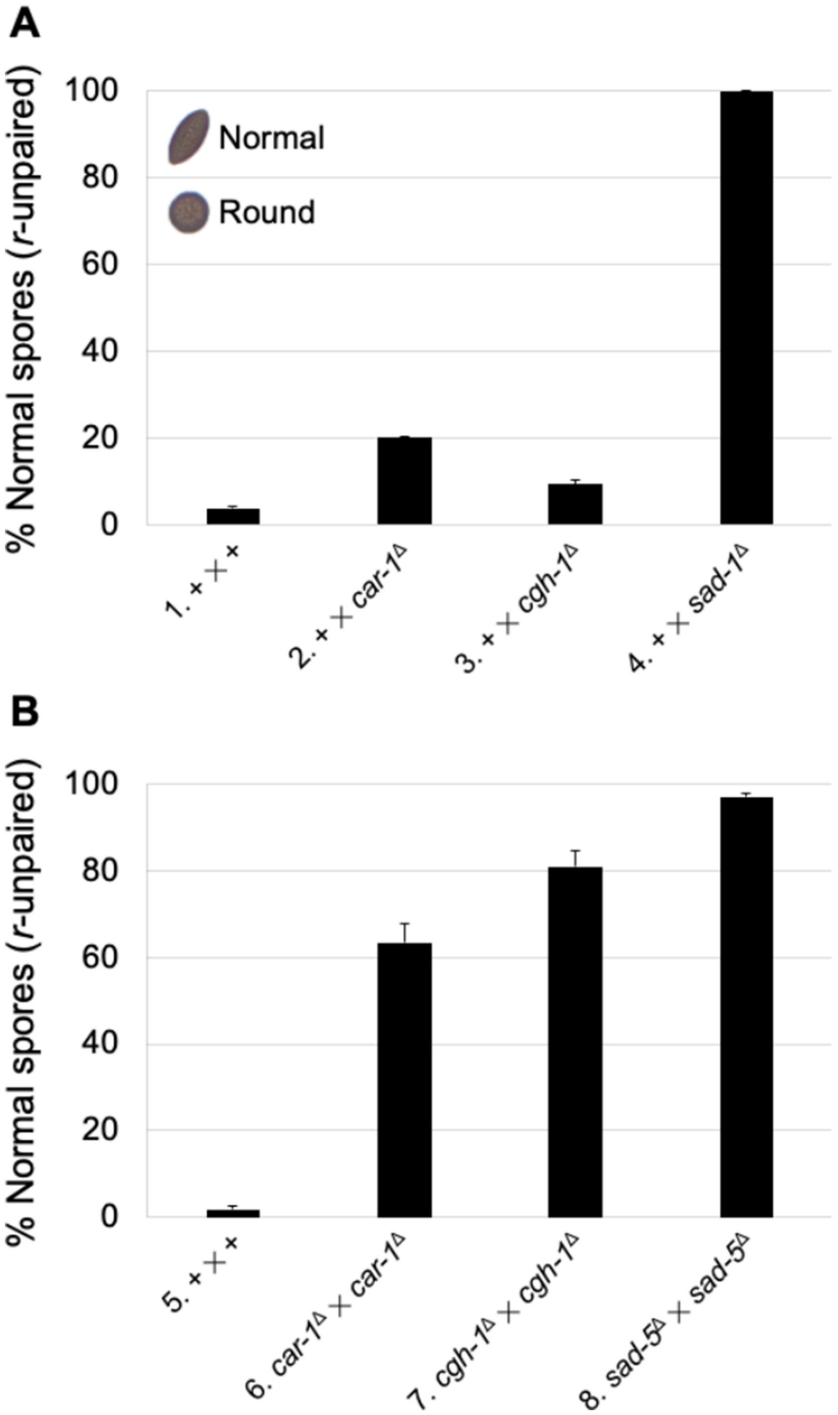
*car-1* and *cgh-1* are involved in MSUD. In *Neurospora*, a normal cross yields American football-shaped ascospores. Here, crosses heterozygous for *r*^Δ^ were tested. In an MSUD-proficient background, an unpaired *r*^+^ is silenced and nearly all of the progeny are round (with 2–4% football; crosses 1 and 5). (A) However, if one parent of an *r*-unpaired cross is *car-1*^Δ^ or *cgh-1*^Δ^, the percentage of normal progeny goes up noticeably (with 20 and 9% football; crosses 2 and 3, respectively), suggesting that MSUD is impaired. (B) If both parents are *car-1*^Δ^ or *cgh-1*^Δ^, progeny are predominantly normal (with 63 and 81% football; crosses 6 and 7, respectively). Also shown here are results for *sad-1*^Δ^ and *sad-5*^Δ^ (with 100 and 97% football; crosses 4 and 8, respectively), two standard MSUD suppressors used as positive controls (Hammond *et al.* 2013b). (1) F2-29 × P9-42. (2) F2-29 × P24-64. (3) F2-29 × P11-67. (4) F2-29 × P3-25. (5) F2-29 × P9-42. (6) F8-01 × P26-34. (7) F7-18 × P26-32. (8) F5-36 × P17-70.

Because RNA granules are linked to post-transcriptional regulation (Anderson and Kedersha 2009; Tian *et al.* 2020), we asked whether CAR-1 and CGH-1 are important for MSUD in *Neurospora*. *Neurospora* normally produces American football-shaped spores. In an *r*^+^ × *r*^**Δ**^ cross, the *round spore* gene is unpaired and silenced (Shiu *et al.* 2001), leading to the production of mostly round spores (or ∼2–4% football; Figure 5, A and B). This “silenced” phenotype could be reversed (or partially reversed) by having an MSUD gene deletion in one or both parents (Hammond *et al.* 2013b). When one parent of an *r*-unpaired cross contains a *car-1*^**Δ**^ or *cgh-1*^**Δ**^ allele, 20 and 9% of the progeny are of football-shaped, respectively, suggesting that the two deletion mutations act as semidominant suppressors of MSUD (Figure 5A). In crosses homozygous for *car-1*^**Δ**^ or *cgh-1*^**Δ**^, the figures go up to 63 and 81%, respectively (Figure 5B). These results suggest that the two RNA granule proteins are involved in the MSUD pathway.

### CAR-1 and CGH-1 interact with components of the MSUD machinery

**Figure 6:**
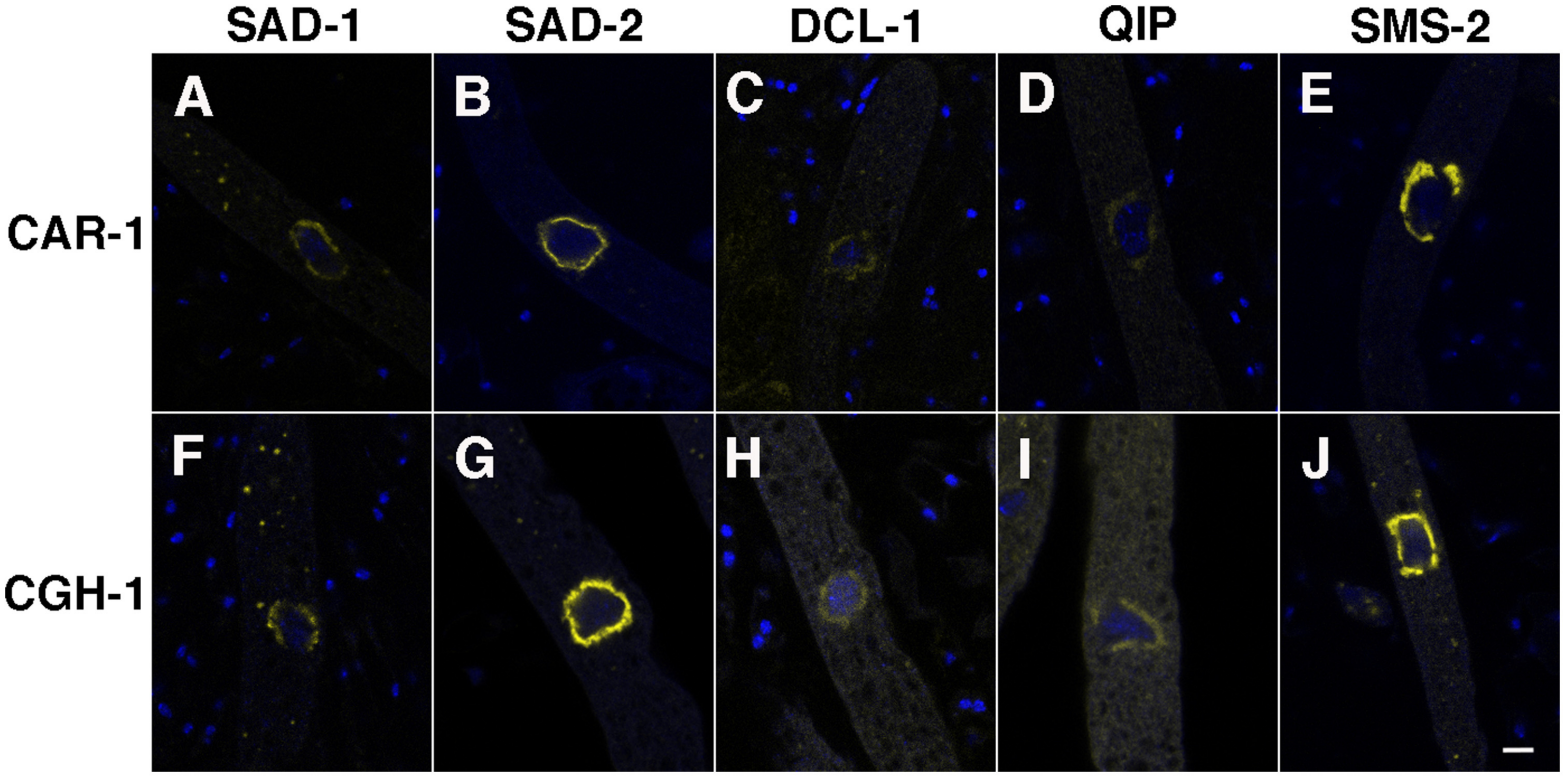
CAR-1 and CGH-1 interact with SAD-1 (RdRP), SAD-2 (scaffold protein), DCL-1 (Dicer), QIP (exonuclease), and SMS-2 (Argonaute). Micrographs illustrate prophase asci expressing (A) *yfpc-car-1* and *yfpn-sad-1* (P26-26 × P26-27), (B) *yfpc-car-1* and *yfpn-sad-2* (P25-31 × P25-32), (C) *yfpc-car-1* and *yfpn-dcl-1* (P26-22 × P26-23), (D) *yfpc-car-1* and *yfpn-qip* (P26-24 × P26-25), (E) *yfpc-car-1* and *yfpn-sms-2* (P25-27 × P25-28), (F) *yfpn-cgh-1* and *yfpc-sad-1* (P26-18 × P26-19), (G) *yfpn-cgh-1* and *yfpc*-*sad-2* (P26-28 × P26-29), (H) *yfpn-cgh-1* and *yfpc-dcl-1* (P26-20 × P26-21), (I) *yfpn-cgh-1* and *yfpc-qip* (P26-30 × P26-31), and (J) *yfpn-cgh-1* and *yfpc-sms-2* (P25-29 × P25-30). Bar, 5 μm.

The perinuclear localization of CAR-1 and CGH-1 suggests that they could be linked to MSC, the silencing complex surrounding the nucleus. Indeed, as shown by BiFC, CAR-1 and CGH-1 interact with known MSC factors (SAD-1, SAD-2, DCL-1, QIP, and SMS-2; Figure 6). These interactions are consistent with the notion that CAR-1 and CGH-1 are involved (directly or indirectly) in the meiotic silencing of target mRNAs.

### The absence of MSUD does not hinder visible P-body formation

**Figure 7:**
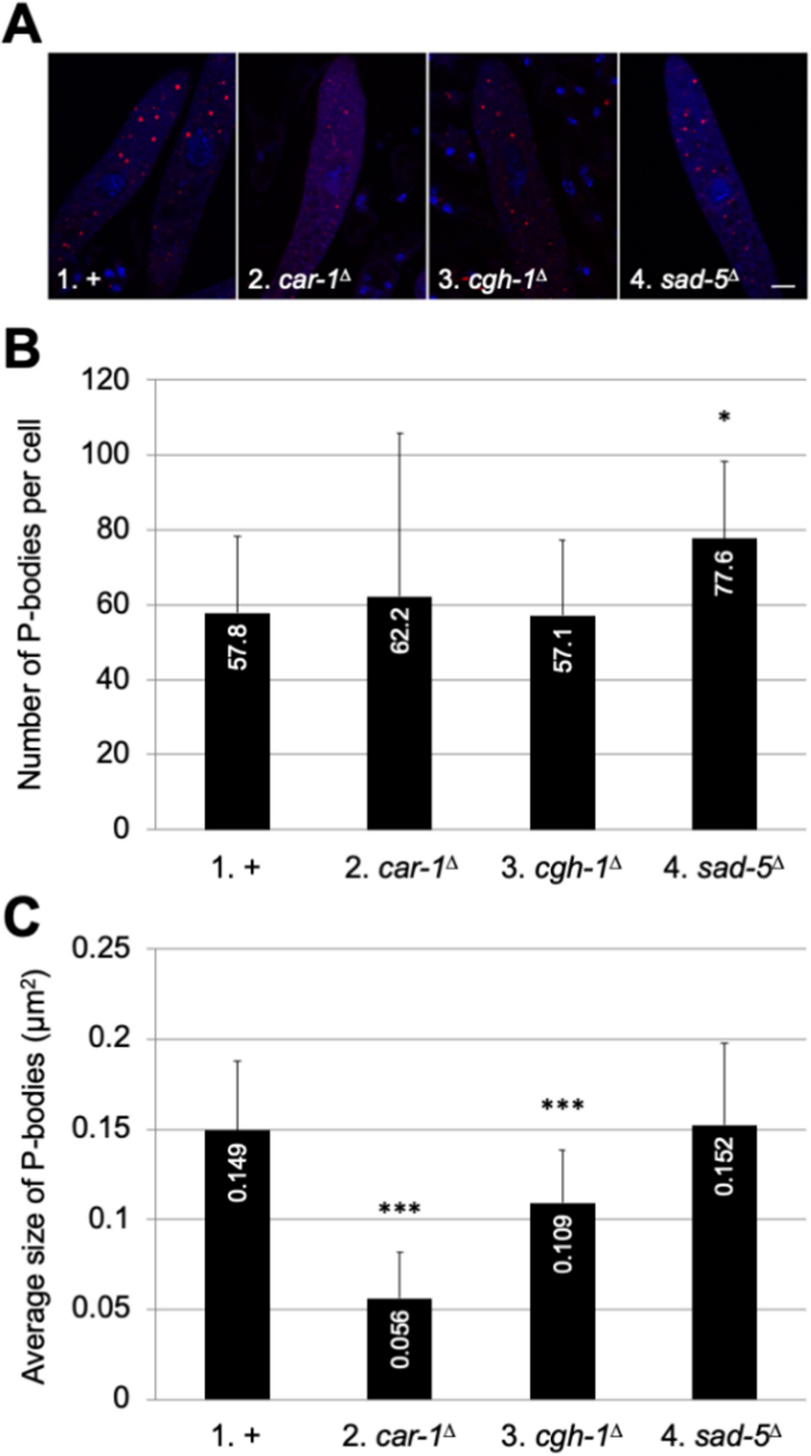
Visible P-body formation in various asci. (A) Micrographs illustrate prophase asci expressing *mCherry-dcap-2* in an MSUD-proficient cross (1, F8-10 × P26-36), a *car-1*-null cross (2, P27-18 × P27-19), a *cgh-1*-null cross (3, F9-01 × P27-20), and a *sad-5*-null cross (4, F8-11 × P26-37). Bar, 5 μm. (B) When compared to the control (+), none of the mutant crosses have a substantially lower P-body count. (C) The average size of P-bodies is markedly lower in a *car-1*^Δ^ or *cgh-1*^Δ^ background. For + *versus* mutant, * indicates *P *<* *0.05 and *** indicates *P *<* *0.001.

In mammals, siRNA-mediated silencing induces P-body assembly (Lian *et al.* 2007). We asked if the absence of MSUD would diminish the production of visible P-bodies in *Neurospora* asci. SAD-5 is crucial for the production of siRNAs, and MSUD becomes nonfunctional in its absence (Hammond *et al.* 2013b). As seen in Figure 7, the absence of SAD-5 (and MSUD) does not reduce visible P-body formation (in terms of number and average size).

Because CAR-1 and CGH-1 are P-body factors (Jain and Parker 2013), we examined whether their absence would affect P-body formation in *Neurospora*. While the number of visible P-bodies is not obviously reduced in a cross devoid of *car-1* or *cgh-1*, their average size is (Figure 7).

## Discussion

In this study, we have demonstrated the presence of P-bodies during sexual development in *Neurospora*. P-body formation could be a consequence of RNA silencing (Eulalio *et al.* 2007b). The silencing machinery could promote mRNA degradation and trigger the assembly of decay mRNA-protein complexes that aggregate into P-bodies (Anderson and Kedersha 2009). We did not observe a reduced P-body production in SAD-5’s absence, which affects siRNA biogenesis during MSUD. If MSUD indeed involves P-bodies, one possibility is that visible P-body formation can be saturated by other pathways in MSUD’s absence. Alternatively, MSUD may mainly involve submicroscopic ribonucleoprotein complexes (Leung and Sharp 2013). Aggregation into a visible entity may not confer additional advantages and is often not essential for RNA granule functions (Anderson and Kedersha 2009; Thomas *et al.* 2011; Leung and Sharp 2013).

CAR-1 and CGH-1 are involved in MSUD. The absence of either protein markedly reduces the severity of meiotic silencing. CAR-1 and CGH-1 are found in cytoplasmic P-bodies as well as the perinuclear region (the center of MSUD activity). The latter localization brings to mind the case in *C. elegans*, in which the two proteins are components of perinuclear P granules (germline structures important for post-transcriptional regulation; Sundby *et al.* 2021). The fact that RNA surveillance often happens in the perinuclear region is hardly a coincidence (also see examples in mammals and flies; Meikar *et al.* 2011; Kloc *et al.* 2014). It could provide an environment in which exported RNAs can meet up with their developmental regulators and have their fates effectively controlled (Voronina 2013; Decker *et al.* 2015).

Using BiFC, we have shown that CGH-1 has interaction with the SMS-2 Argonaute. This is similar to the situation in human cells, in which a CGH-1 homolog (RCK) interacts with Argonaute proteins (to mediate translation repression; Chu and Rana 2006). It is possible that certain complex formations are conserved among different post-transcriptional networks. Interestingly, CAR-1 has also been shown to interact with the SMS-2 Argonaute in this study.

Although many components of the meiotic silencing machinery have been revealed, the final fates of MSUD-targeted mRNAs remain unclear. Our current model proposes that the SMS-2 Argonaute uses siRNAs to guide the slicing of complementary mRNAs (presumably in the perinuclear region). It is conceivable that targeted mRNA slicing is not 100% efficient and that unsliced (and possibly sliced) mRNAs are associated with certain RNA granule proteins for degradation, repression, and/or storage. CAR-1 and CGH-1 family proteins are presumed to be translation repressors and/or decapping activators (Eulalio *et al.* 2007a; Jain and Parker 2013; Zeidan *et al.* 2018). In the absence of CAR-1 or CGH-1, unsliced mRNAs could be inadvertently released for translation, allowing some expression of unpaired genes. Although the above hypothesis could explain our observations thus far, other possibilities abound. For example, CAR-1 and CGH-1 could enhance the activity of the SMS-2 Argonaute and/or other MSUD factors (*i.e.*, their usual P-body functions are not involved in MSUD). Future work on these and other RNA granule components will shed light on how they regulate gene expression and diverse processes.

## Data availability

Strains are available upon request. Supplementary material is available at figshare: https://doi.org/10.25387/g3.14575689.
