# Corrigendum to: Involvement of RNA granule proteins in meiotic silencing by unpaired DNA

**DOI:** 10.1093/g3journal/jkab326

**Published:** 2021-10-06

**Authors:** Hua Xiao, Michael M Vierling, Rana F Kennedy, Erin C Boone, Logan M Decker, Victor T Sy, Jackson B Haynes, Michelle A Williams, Patrick K T Shiu

G3 Genes|Genomes|Genetics, Volume 11, 2021. https://doi.org/10.1093/g3journal/jkab179

In the originally published version of this manuscript (*i.e.*, the corrected proof), a strain not used in the study (P26-35) was inadvertently included in Table 1. This strain has now been deleted. 

